# Quality of Life of Ostomates with the Selected Factors in a Selected Hospital of Delhi with a View to Develop Guidelines for the Health Professionals

**DOI:** 10.4103/0973-1075.58455

**Published:** 2009

**Authors:** Aditi Sinha, Harinderjeet Goyal, Shailja Singh, Shiv Pratap Singh Rana

**Affiliations:** Dr. BR Ambedekar Institute Rotary Cancer Hospital, AIIMS, New Delhi, India; 1 RAK, College of Nursing, New Delhi, India; 2 Department of Anaesthesia, Dr. BR Ambedekar Institute Rotary Cancer Hospital, AIIMS, New Delhi, India

**Keywords:** Colostomy, Ostomates, Quality of life

## Abstract

**Background:**

A correlational survey to assess the quality of life (QoL) of ostomates with selected factors in a selected hospital of Delhi and to develop guidelines for the health professionals to improve QoL of ostomates was undertaken during the year 2005-2007. The objectives of the study were as follows: (1) To assess QoL of ostomates (2) To identify the factors associated with QoL of ostomates (3) To seek relationship between selected factors and QoL of ostomates (4) To develop guidelines for the health professionals to improve QoL of ostomates.

**Materials and Methods:**

The corelational survey was conducted on 50 ostomates from Surgical Oncology OPD of B.R.A. Institute Rotary Cancer Hospital, AIIMS, Delhi. Purposive sampling technique was employed to select the sample subjects. Structured interview schedule was prepared. Guidelines were prepared for health professionals to improve the quality of life of ostomates.

**Result:**

Majority of the ostomates possessed best quality of life. There was a significant association between QoL score of ostomates with age, sex, duration of surgery, education, income, and occupation. There was no significant association between QoL scores of ostomates and marital status and type of ostomy.

**Conclusion:**

This study conclude that nurses have a great role to play in the physical, psychological, economical, social, familial, and sexual aspects in the care of ostomates and to offer psychological support and empathy, to reinforce coping skills to promote an optimal QoL.also she has a great role to influence and educate all the aspects of care to the patients and their relatives. Their is a need to develop staff development program for nursing personnel in the clinical area in healthcare system.

## INTRODUCTION

The quality of life (QoL) is of central concern in evaluative research; improved QoL is probably the most desirable outcome of all healthcare policies.[1] QoL is defined as a degree of satisfaction or dissatisfaction felt by people with various aspects of their lives. QoL includes both conditions of life and the experiences of life.[2]

The individual with colostomy or ileostomy undergoes a complex treatment with a wide range of adjustments affecting the individual's social and psychological functioning. QoL is an outcome measure worth considering for achieving a holistic approach for measuring the impact of treatment to maximize the QoL of the individual.[3]

A person who is living with the dreadful duo of cancer and colostomy has to cope up with the emotional trauma of his new body image and the daily care of his stoma. In such a situation, the role of nurse as an emotional supporter and patient educator becomes especially important.[4] Guidelines to help the patient to cope with stoma will help the health professionals to provide counseling. A correlational survey was undertaken to assess QoL of ostomates with selected factors in a selected hospital of Delhi to develop guidelines for the health professionals to improve QoL of ostomates.

## MATERIALS AND METHODS

The conceptual framework adopted for the study was based on Orem's Self Care Model. The research approach adopted for the study was correlational survey. The study was conducted on 50 ostomates from Surgical Oncology OPD of B.R.A. Institute Rotary Cancer Hospital, AIIMS, Delhi.

A structured interview schedule was developed for data collection. Structured interview schedule consists of three parts. Part IA dealt with demographic data; Part IB dealt with ostomy-related information; and Part II dealt with the assessment of QoL. Tool was validated by nine experts from the field of surgical oncologists, enterostomal therapists, psychiatry, etc. Reliability of the tool was established by using Cronbach Alpha formula, and Alpha coefficient was found to be 0.74.

Colostomates and ileostomates were interviewed who fulfilled the following sampling criteria:

- Minimum 6 weeks post-surgery subjects
- Subjects in the age group of 18-59 years
- Subjects who were willing to participate in the study

A guideline was prepared for health professionals to improve QoL of ostomates and was validated by experts. Data collected was analyzed using both descriptive and inferential statistics based on the objectives in terms of frequencies, percentage, mean, and Chi-square and *t*-test.

## RESULTS

### Description of sample characteristics

**Figure 1:**
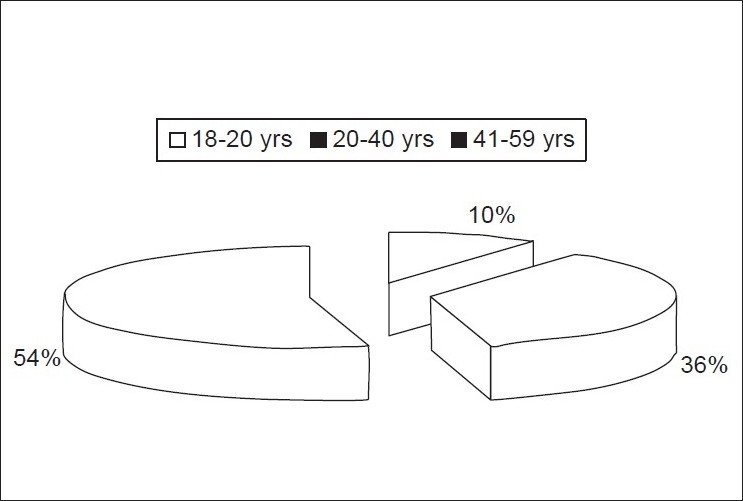
Pie chart showing the percentage of ostomates according to age group
- Most (70%) of ostomates were males [Figure 2]

- (54%) of ostomates were in the age group of 41-59 years [Figure 1]

**Figure 2:**
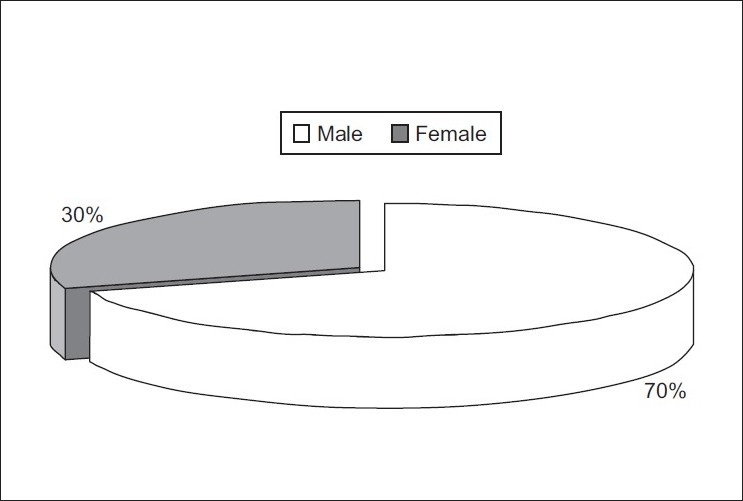
Pie chart showing the distribution of ostomates according to sex
- Majority (70%) of ostomates had colostomy
- Majority (76%) of ostomates had 0-10 years duration of ostomy
- 34% of ostomates had a change in their clothing style because of ostomy
- Majority (66%) of ostomates had a change in their diet because of ostomy
- 38% of ostomates had problem while traveling due to ostomy
- All (100%) of ostomates felt comfortable with their ostomy care.

### Findings related to quality of life scores of ostomates

The range of QoL score was 50150.

**Figure 3:**
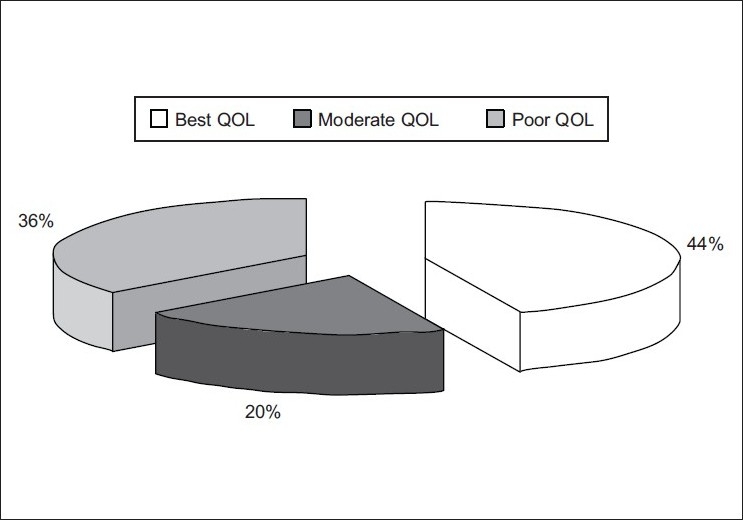
Pie chart showing the percentage of ostomates with different levels of quality of life

- Majority (44%) of ostomates possessed best QoL, and 36% of the ostomates possessed poor QoL and with least (20%) possessed moderate QoL [Figure 3].

### Findings related to relationship of quality of life scores with selected background factors

- There was a significant association between QoL score of ostomates with age, sex, duration of surgery, education, income, and occupation [Table 1].

**Table 1:** Relationship with selected factors with quality of life scores of ostomates (N = 50)

Selected factors	Best quality of life scores	Moderate and poor quality of life scores	df	Chi-square value	S/NS
Age					
Below 40 years	16	7	1	5.24*	S
Above 40 years	10	17		
Sex					
Male	24	11	1	5.34*	S
Female	5	10		
Marital status					
Single	3	5	1	0.26	NS
Married	20	22		
Educational level					
Below primary	1	6	3	11.79*	S
Secondary	3	10		
Graduate	14	9		
Postgraduate	6	1		
Income					
Below 5000 per month	10	17	1	3.92*	S
Above 5000 per month	15	8		
Occupation					
Employed	12	27	1	3.91*	S
Unemployed	7	4		
Duration of surgery					
Below 10 years	15	24	1	4.58*	S
Above 10 years	8	2	1	0.44	NS
Type of ostomy					
Colostomy	15	15		
Ileostomy	8	7		

χ^2^ value is significant at 0.05 level; df (1) = 3.84 *P* > 0.05; df (3) = 7.81 *P* > 0.05;

* Significant at 0.05 level of significance
- There was no significant association found in QoL scores of ostomates with marital status and type of ostomy.
- There was no significant difference between mean QoL score of colostomates with mean QoL score of ileostomates.

## DISCUSSION

No similar study is done. But various reviews are available based on which we can deduce that stoma can affect QoL of individuals. White and Hunt[5] suggest that stoma formation can result in psychological morbidity. Black[6] suggests that stoma formation usually has a negative impact on a person's QoL and affects life styles in a number of ways. Baxter and Salter[7] identify ways in which nurses can help stoma patients come to terms with their diagnosis and prognosis, adapting to life with a stoma, teaching practical skills in caring for their stoma and addressing issues around family support networks, employment, body image, and sexuality. Collet[8] suggested that poor psychological adjustment to stoma surgery has been shown to correlate to depression and predict death later on. If the nurses provide opportunities for open and general communication for patients with stoma, then it is more likely that the feelings will be enhanced and will help the patient to accept their new body image and improve their QoL.

Study is conducted and identified the factors that affect QoL of ostomates and guideline is prepared that will help the healthcare workers to improve QoL of ostomates. Nurses have a great role to play in the physical, psychological, economical, social, familial, and sexual aspects in the care of ostomates and to offer psychological support and empathy, to reinforce coping skills to promote an optimal QoL as she spends ample amount of time with ostomates; so she has a great role to influence and educate all the aspects of care to the patients and their relatives. Learning opportunities should be given to the nursing students in encouraging clients to restore their QoL, which is the need of the day. Earlier the emphasis was only on curing the symptoms, but now the emphasis is on maximizing QoL. The staff development program for nursing personnel in the clinical area is inadequate in the existing healthcare system. The nurse administrators should organize continuing education program to update the knowledge of nurses so that they can assist the ostomates to improve their QoL. Research studies conducted by Indian nurses in this area are very few. It is high time that all nursing personnel join their hands to provide scientifically tested materials or programs toward the QoL assessment of the ostomates and improve their QoL.

## CONCLUSION

- Majority of above 40 years of age group had ostomy
- Majority of the ostomates had colostomy
- Majority of the ostomates possessed best QoL
- All of the ostomates felt comfortable with ostomy care
- There was a significant association between QoL score of ostomates with age, sex, duration of surgery, education, income, and occupation
- There was no significant association found in QoL scores of ostomates with marital status and type of ostomy.
